# Etymologia: *Paracoccidioides*

**DOI:** 10.3201/eid2709.210461

**Published:** 2021-09

**Authors:** Lucas Nojosa Oliveira, Patrícia de Sousa Lima

**Affiliations:** Faculdade Estácio de Sá de Goiás, Goiás, Brazil (L.N. Oliveira); Universidade Estadual de Goiás, Goiás (P. de Sousa Lima)

**Keywords:** Paracoccidioides, Paracoccidioides brasiliensis, Paracoccidioides lutzi, fungi, dimorphic fungus, mycelium form, yeast form, fungal infections, soil, inhalation, Adolpho Lutz, Latin America

## Paracoccidioides [p′a ɾə kok-sidʺe-oiʹ d′ez]

**Figure 1:**
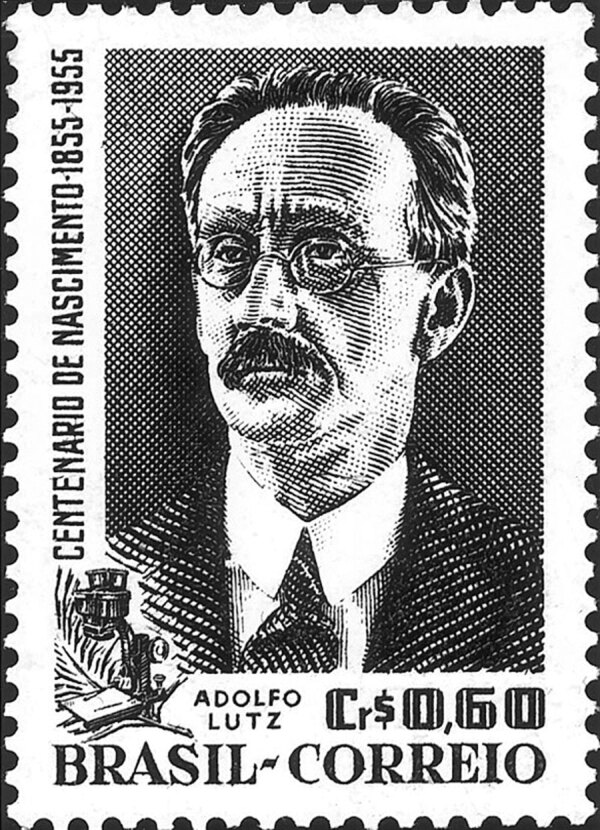
Adolfo Lutz (1855‒1940). Unknown author, Wikimedia Commons

From the Greek (*para*/παρά + *kokkis* [coccidia]), Adolpho Lutz (Figure 1) described *Paracoccidioides* in 1908. After analysis of oral and cervical lymph node lesions from infected patients, Lutz initially believed that he had detected *Coccidioides*. However, more extensive analysis showed that he had detected another fungus. Because of morphologic and clinical disease similarities, the name *Paracoccidioides* was suggested. The prefix para (near) indicates its similarity with *Coccidioides*.

**Figure 2:**
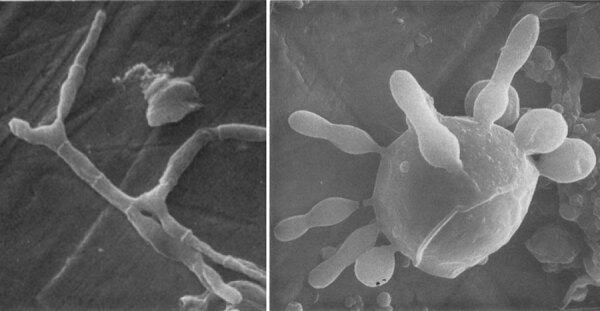
*Paracoccidioides brasiliensis* mycelium cells (left) and multibudding yeasts (right) by scanning electron microscopy.  Original magnifications ×1,500 for the left panel and ×3,000 for the right panel. Image adapted from Vieira e Silva et al. 1974.

*Paracoccidioides* is a thermally dimorphic fungus (Figure 2). It grows as an infective mycelium form (at 18°C–23°C) or a parasitic multibudding yeast form (at 35°C–37°C). It is composed of 2 species: *P. brasiliensis* and *P. lutzi*. They are the etiologic agents of paracoccidioidomycosis. This systemic infection is endemic to Latin America (southern Mexico to northern Argentina). The highest number of cases are found in Brazil, Colombia, and Venezuela. *Paracoccidioides* conidia and mycelia are found in soil and transmitted by inhalation.
